# An Interprofessional Team for Disease Modifying Therapy in Alzheimer’s Disease Implementation

**DOI:** 10.1002/alz.091725

**Published:** 2025-01-09

**Authors:** Katherine W. Turk, Mark D Knobel, Alexandra Nothern, Garrett Friedman, Hannah Noah, Brendan Campbell, Diana Anderson, Andreas Charidimou, Andrew Mills, Vanessa Coronel, Nacha Pierre, Beverly Reynolds, Caroline Wagner, Leanne Varga, John Roefaro, Laura Triantafylidis, Andrew E. Budson

**Affiliations:** ^1^ Boston University Alzheimer’s Disease Research Center, Boston, MA USA; ^2^ Boston University Chobanian & Avedisian School of Medicine, Boston, MA USA; ^3^ VA Boston Healthcare System, Jamaica Plain, MA USA; ^4^ VA BOSTON HEALTH CARE SYSTEM, BOSTON, MA USA; ^5^ Boston University School of Medicine, Boston, MA USA; ^6^ VA Boston Healthcare System, Boston, MA USA; ^7^ VA Boston Healthcare System, Jamaica Plai, MA USA

## Abstract

**Background:**

Lecanemab and other new amyloid‐targeting immunotherapies for Alzheimer’s disease show great promise but, may also pose significant risk for patients. To facilitate the implementation and monitoring of lecanemab infusions at our tertiary medical center, we convened an interprofessional team. The team created a number of resources including patient handouts and medical documentation templates as well as systems and processes. As the first VA in the country, and one of the first clinical settings in the country overall to begin Lecanemab infusions, it is our intent to widely share the resources and processes developed as well as share initial clinical experiences.

**Methods:**

An interprofessional team at VA Boston Healthcare System (VABHS) (Disease Modifying Therapy in Alzheimer’s Disease, DMTAD group): behavioral neurologology attendinds and fellows, neuropsychiatry fellows, geriatricians, neuroradiologist, infusion nurses, geriatric clinical pharmacists, and a physician assistant was assembled and began meeting weekly for one hour. The team’s initial objective was to streamline processes and systems for initiation of lecanemab treatment as well as assessment, review, and monitoring of patients from memory disorders and geriatrics clinics for potential treatment based on VA criteria for use. Responsibilities of the group also included placing medication orders, routinely reviewing imaging, and monitoring for any medication‐related side effects and adverse reactions patients may have experienced in the preceding week.

***Results and Clinical Experiences to Date***: Since infusions began in October 2023 16 patients have completed full screening evaluation for including amyloid PET, APOE testing, MRI brain within 3 months, and baseline lab work.

8 were ineligible for Lecanemab: blood thinners (6), APOE 4 homozygosity (2).

20 additional patients undergoing screening work up for eligibility currently.

8 patients are actively completing infusions: 4 have experienced mild to moderate side effects including chills and flu like sensations after infusions 1 or 2.

4 have tolerated without side effects. No patients have experienced ARIA to date.

**Conclusions:**

An interprofessional team is effective at screening and monitoring potential Lecanemab patients at a tertiary medical center and overall screened patients are safely tolerating infusions in a real world setting.

